# Role of soluble triggering receptor expressed on myeloid cells-1 for diagnosing ventilator-associated pneumonia after cardiac surgery: an observational study

**DOI:** 10.1186/1471-2261-13-107

**Published:** 2013-12-01

**Authors:** Alessandra K Matsuno, Ana PCP Carlotti

**Affiliations:** 1 Division of Pediatric Critical Care Medicine, Department of Pediatrics, Ribeirão Preto Medical School, University of São Paulo, Ribeirão Preto, Brazil; 2 Department of Pediatrics, Hospital das Clínicas, Ribeirão Preto Medical School, University of São Paulo, Avenida dos Bandeirantes, 3900, 14049-900 Ribeirão Preto, SP, Brazil

**Keywords:** Triggering receptor expressed on myeloid cells-1, Ventilator-associated pneumonia, Diagnosis, Cardiac surgery, Postoperative

## Abstract

**Background:**

The diagnosis of ventilator-associated pneumonia (VAP) is a challenge, particularly after cardiac surgery. The use of biological markers of infection has been suggested to improve the accuracy of VAP diagnosis. We aimed to evaluate the usefulness of soluble triggering receptor expressed on myeloid cells (sTREM)-1 in the diagnosis of VAP following cardiac surgery.

**Methods:**

This was a prospective observational cohort study of children with congenital heart disease admitted to the pediatric intensive care unit (PICU) after surgery and who remained intubated and mechanically ventilated for at least 24 hours postoperatively. VAP was defined by the 2007 Centers for Disease Control and Prevention criteria. Blood, modified bronchoalveolar lavage (mBAL) fluid and exhaled ventilator condensate (EVC) were collected daily, starting immediately after surgery until the fifth postoperative day or until extubation for measurement of sTREM-1.

**Results:**

Thirty patients were included, 16 with VAP. Demographic variables, Pediatric Risk of Mortality (PRISM) and Risk Adjustment for Congenital Heart Surgery (RACHS)-1 scores, duration of surgery and length of cardiopulmonary bypass were not significantly diferent in patients with and without VAP. However, time on mechanical ventilation and length of stay in the PICU and in the hospital were significantly longer in the VAP group. Serum and mBAL fluid sTREM-1 concentrations were similar in both groups. In the VAP group, 12 of 16 patients had sTREM-1 detected in EVC, whereas it was undetectable in all but two patients in the non-VAP group over the study period (p = 0.0013) (sensitivity 0.75, specificity 0.86, positive predictive value 0.86, negative predictive value 0.75, positive likelihood ratio (LR) 5.25, negative LR 0.29).

**Conclusion:**

Measurement of sTREM-1 in EVC may be useful for the diagnosis of VAP after cardiac surgery.

## Background

Ventilator-associated pneumonia (VAP) is the most common nosocomial infection in children following cardiac surgery in Brazil and European countries [1-3]. Its reported incidence ranges from 17.1 to 42.8 episodes per 1000 ventilator-days and it is associated with increased duration of mechanical ventilation, increased length of pediatric intensive care unit (PICU) and hospital stay and higher mortality rates [1,2,4].

The diagnosis of VAP is a major challenge in patients undergoing cardiac surgery, because the systemic inflammatory response syndrome elicited by surgical trauma and the interaction of blood with the cardiopulmonary bypass (CPB) circuit may induce symptoms that are similar to those caused by systemic infections [5]. In addition, imaging studies may show pulmonary opacities caused by fluid overload, capillary leak, pulmonary contusion resulting from surgical manipulation, atelectasis or alveolar hemorrhage, which are comparable to those found in patients with pneumonia. Although in critically ill patients with severe bacterial infections early initiation of appropriate antibiotic therapy is crucial for survival, in patients with the systemic inflammatory response syndrome associated with non-infectious processes unnecessary treatment with antibiotics may lead to increased bacterial resistance and increased costs, with no treatment benefit. This highlights the importance of making an accurate diagnosis of VAP.

The gold standard method for diagnostic confirmation of VAP is lung tissue examination and culture [6,7], but its clinical applicability is limited. The clinical and radiologic criteria recommended by the Centers for Disease Control and Prevention (CDC) have limited specificity whereas the microbiological criteria lack specificity and sensitivity compared with autopsy lung culture [6]. Thus, the use of biological markers of infection as the triggering receptor expressed on myeloid cells (TREM)-1 has been suggested to improve the accuracy of the diagnosis of VAP [8-10].

TREM-1 is a transmembrane glycoprotein expressed on neutrophils, macrophages and monocytes that amplifies the inflammatory response. Its expression by the effector cells is upregulated in tissues infected by bacteria and fungi [11]. The activation of TREM-1 stimulates the production of proinflammatory cytokines and induces neutrophil degranulation and oxidative burst [12]. Moreover, infection induces the release of a soluble form of this receptor from the membranes of activated phagocytes (sTREM-1) which can be measured in body fluids and may be a useful diagnostic tool [13]. It has been shown that plasma sTREM-1 concentrations are increased in patients with sepsis [14]. In addition, elevated levels of TREM-1 have been found in bronchoalveolar lavage (BAL) fluid and in the fluid collected from the ventilator expiratory trap, the exhaled ventilator condensate (EVC), in critically ill adults with VAP [8-10]. However, increased plasma concentrations of TREM-1 have also been reported in patients with the systemic inflammatory response syndrome secondary to cardiac surgery with CPB and after cardiac arrest, without infection [15]. Nevertheless, there are no published data on the role of sTREM-1 in the diagnosis of VAP in patients undergoing cardiac surgery. To address this question, we performed serial measurements of sTREM-1 concentrations in serum, modified non-bronchoscopic bronchoalveolar lavage (mBAL) fluid and EVC samples in intubated and mechanically ventilated children following cardiac surgery. We sought to evaluate whether the use of sTREM-1 could improve the accuracy of VAP diagnosis after cardiac surgery.

## Methods

### Study design and patients

This was a prospective cohort study conducted in a PICU of a tertiary-care university hospital in Brazil. The study was approved by the Research Ethics Board of Hospital das Clínicas of Ribeirão Preto Medical School, University of São Paulo (#1499/2008) and written informed consent was obtained from the patients’ parents. Children with congenital heart disease admitted to the PICU following cardiac surgery from May 2008 to April 2009 and who remained intubated and mechanically ventilated for at least 24 hours after surgery were eligible for the study. Exclusion criteria were a preoperative diagnosis of nosocomial pneumonia and/or sepsis, death in the first 24 postoperative hours and refusal to participate.

Patients received prophylactic antibiotics for 48 hours from the induction of anesthesia with intravenous cefazolin (40 mg/kg every 8 hours) and amikacin (7.5 mg/kg every 12 hours). Patients with a history of hospitalization for more than 72 hours, ICU admission or previous use of antibiotics received intravenous vancomycin (20 mg/kg at the induction of anesthesia followed by 10 mg/kg every 6 hours) and amikacin (7.5 mg/kg every 12 hours).

Patients who underwent CPB received a bolus dose of 30 mg/kg of methylprednisolone or 10 mg/kg of hydrocortisone at CPB start. Modified ultrafiltration was not performed at the end of CPB.

The diagnosis of VAP was made independently by the PICU physician and an infection control practitioner by the 2007 CDC criteria, which considered that no minimum period of time was needed for the ventilator to be in place to diagnose VAP. According to the time of onset, VAP was categorized as early, when it occurred within 1 to 4 days of mechanical ventilation, and late, when it occurred after 4 days of ventilation [6].

### Data collection

Demographic and clinical data were collected from patients’ health records. Procedure complexity was stratified according to Risk Adjustment for Congenital Heart Surgery (RACHS)-1 score [16] and severity of illness was assessed by Pediatric Risk of Mortality (PRISM) score between 8 and 24 hours following PICU admission [17].

Blood, mBAL fluid and EVC were collected daily, starting immediately after surgery (iPO) until the fifth postoperative day (POD5) or until the child was extubated for measurement of sTREM-1 by a sandwich enzyme-linked immunosorbent assay (ELISA – kit Quantikine – R&D Systems, Minneapolis, MN, USA). Blood samples were collected from arterial lines inserted before surgery for blood pressure monitoring. mBAL fluid was collected using a feeding tube, which was introduced down the endotracheal tube until resistance was felt. Then, using a syringe, 1 ml/kg of normal saline (maximum 20 ml) was instilled through the feeding tube followed by a small amount of air to clear the deadspace of the catheter and suction was applied to obtain the fluid [18]. Quantitative cultures of mBAL fluid were considered positive when the growth of >10^5^ CFU/mL was observed [19]. EVC (approximately 1 ml) was collected from the trap located in the expiratory limb of the ventilator circuit [10]. Nasopharyngeal aspirates were collected from all patients for detection of respiratory viruses by real-time PCR using a standard protocol [20].

### Statistical analysis

Analysis was made using GraphPad Prism 5.0 (San Diego, CA) and SAS 9.2, R 2.15.1 (SAS/STAT® User’s Guide, Version 9.2, Cary, NC: SAS Institute Inc., 2008). Data were expressed as median (range). Patients were grouped according to the presence or absence of VAP. Continuous variables between groups were compared by Mann–Whitney *U* test and categorical variables by Fisher’s exact test. Variables with repeated measurements (serum and mBAL sTREM-1) were analyzed after logarithmic transformation using a linear mixed-effects model where individual measures over time were included as a random factor and group membership and time as fixed factors. Because there were many undetectable values of EVC sTREM-1 concentrations, they were categorized as positive and negative, and analysed by Fisher’s exact test. Association between variables was assessed by Spearman’s correlation. A Receiver Operating Characteristic (ROC) curve was constructed for determination of cut-off value of sTREM-1 in serum, mBAL and EVC for the diagnosis of VAP, considering values on the day of VAP diagnosis for the VAP group and the largest value over the study period for the non-VAP group, since increased concentrations of sTREM-1 could also be related to the systemic inflammatory response to surgery and CPB, and pulmonary congestion. A p value < 0.05 was considered significant.

## Results

### Characteristics of the patients

Over the study period, 145 children with congenital heart disease were admitted to the PICU of our institution after cardiac surgery; 63 met the inclusion criteria. Thirty patients were excluded because consent was not obtained and three because of death in iPO. Thirty patients were included in the study. Their median age was 45 days (range 2 to 4015 days) and their median weight 3.4 kg (range 1.8 to 20 kg). Twenty-six (87%) patients underwent CPB. Seven (23%) patients were left with the sternum open for 2 to 5 days after surgery (median 4 days). Twenty-eight (93%) patients had pulmonary congestion in iPO.

Of the 30 patients included, 16 (53%) developed VAP, which was diagnosed between the first and the fifth postoperative day; the median time to VAP diagnosis was 2 days after surgery. All but one patient had early-onset VAP. The rate of VAP at our PICU during the study period was 22.8 episodes per 1000 ventilator-days. Table 1 shows comparisons between patients with and without VAP. Demographic variables, PRISM and complexity of surgery assessed by RACHS-1 score were similar in both groups. Also, duration of surgery and length of cardiopulmonary bypass were not significantly diferent in patients with and without VAP. However, time on mechanical ventilation and length of stay in the PICU and in the hospital were significantly longer in the VAP group. There were two deaths within 28 days of admission, both in the VAP group, due to septic shock.

**Table 1:** Characteristics of the study groups

***Characteristics***	***Patients with VAP (n = 16)***	***Patients without VAP (n =14)***	***P value***
Age (days)	32 (3–2310)	49 (3–4015)	0.72
Weight (kg)	3.3 (1.8–17.3)	3.8 (2.2–20)	0.83
Male gender	8 (50%)	6 (43%)	0.73
PRISM	11 (4–28)	11 (2–17)	0.23
RACHS-1	4 (2–6)	4 (2–4)	0.65
Use of CPB	14 (88%)	12 (86%)	1
Duration of CPB (minutes)	110 (0–175)	120 (0–160)	0.38
Aortic cross clamp time (minutes)	60 (0–99)	87 (0–113)	0.17
Duration of surgery (minutes)	238 (145–495)	248 (165–410)	0.52
Duration of mechanical ventilation after surgery (days)	8 (3–19)	3 (1–7)	0.0004
PICU length of stay (days)	15 (5–31)	7 (4–16)	0.004
Hospital length of stay (days)	30 (9–218)	12 (7–27)	0.006
Mortality	2 (12.5%)	0	0.48

Legend: Data are reported as median (range) or n (%). PRISM, Pediatric Risk of Mortality; RACHS, Risk Adjustment for Congenital Heart Surgery; CPB, cardiopulmonary bypass; PICU, pediatric intensive care unit.

mBAL culture was positive (considering the cutoff of > 10^5^ CFU/mL) in six patients with VAP, with the growth of the following agents: *Acinetobacter baumannii * (n = 2), *Staphylococcus epidermidis* (n = 1), *Staphylococcus aureus* methicillin-sensitive (n = 1), *Pseudomonas aeruginosa* (n = 1), and *Enterococus faecium (n = 1)*. Blood culture was positive in two patients with VAP, with the growth of *Staphylococcus aureus* methicillin-sensitive (n = 1; the same microorganism grew in mBAL fluid) and *Staphylococcus aureus* methicillin-resistant (n = 1). Bocavirus was detected in nasopharyngeal aspirate in one patient with VAP and rhinovirus was detected in five patients in the VAP group and in three patients in the non-VAP group. Three patients with rhinovirus infection in the VAP group were coinfected with the following agents: *Acinetobacter baumannii*, *S. aureus* methicillin-sensitive and *S. aureus* methicillin-resistant; one patient in the non-VAP group was coinfected with respiratory syncytial virus. mBAL and blood cultures were negative in all patients in the non-VAP group.

### sTREM-1measurements

**Figure 1:**
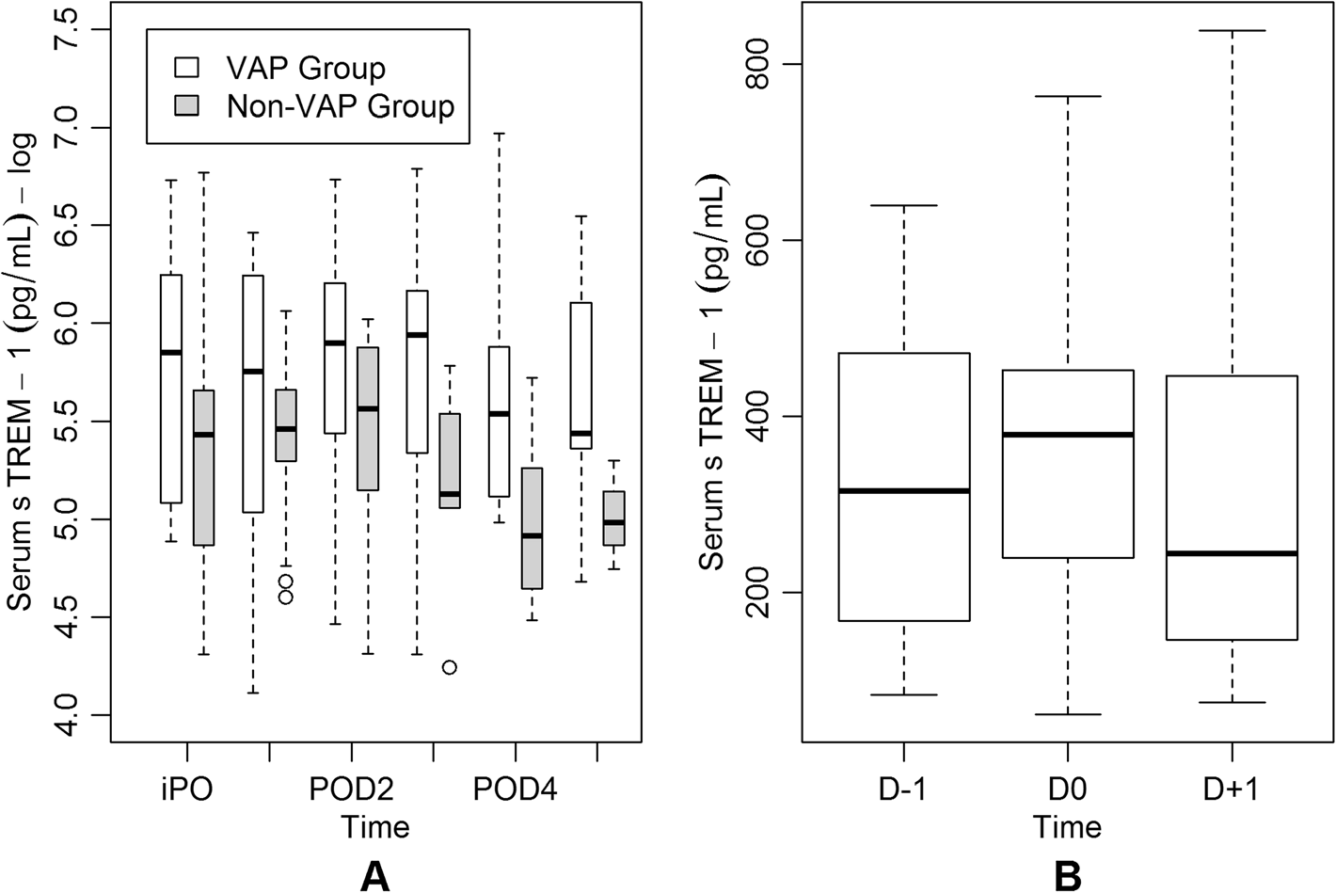
**Serum concentrations of soluble triggering receptor expressed on myeloid cells (sTREM)-1. A.** Daily log-transformed serum concentrations of sTREM-1 in the ventilator-associated pneumonia (VAP) group (white boxes) and in the non-VAP group (boxes with gray shading). **B.** Serum concentrations of sTREM-1 in the VAP group on the day before VAP diagnosis (D-1), on the day of VAP diagnosis (D0) and on the day after VAP diagnosis (D+1). Central lines are medians, boxes are interquartile ranges, and brackets are total range.

There was no significant difference between groups in daily serum sTREM-1 concentrations and values did not change significantly over time in either group (p = 0.25). Peak serum sTREM-1 concentrations were observed on POD3 in patients with VAP (median 379 pg/ml; range 74–888 pg/ml) and POD2 in patients without VAP (median: 267 pg/ml; range: 75–411 pg/ml). In the group with VAP, comparison of serum concentrations of sTREM-1 on the day of VAP diagnosis (D0) with those on the day before (D-1) and on the day after (D+1) the diagnosis of VAP did not show a significant difference (p = 0.71) (Figure 1). Comparison of serum sTREM-1 concentrations on the day of VAP diagnosis with peak serum sTREM-1 concentrations in the non-VAP group also did not show a significant difference (p = 0.16). There was no correlation of serum sTREM-1 peak concentrations with duration of CPB (r = 0.08; p = 0.65) or duration of aortic cross-clamping time (r = -0.07; p = 0.72).

**Figure 2:**
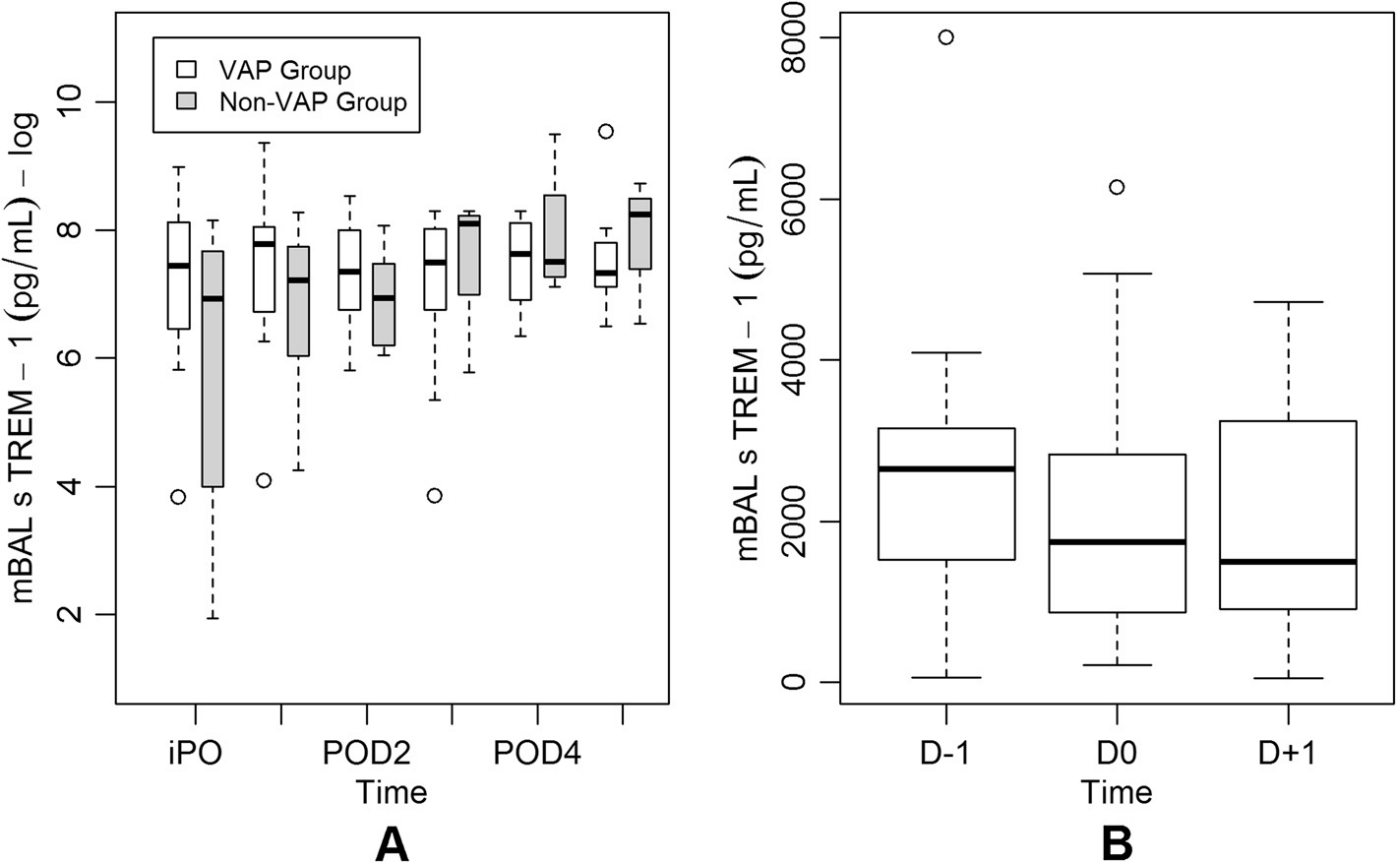
**Concentrations of soluble triggering receptor expressed on myeloid cells (sTREM)-1 in modified non-bronchoscopic bronchoalveolar lavage (mBAL) fluid. A.** Daily log-transformed concentrations of sTREM-1 in mBAL fluid in the ventilator-associated pneumonia (VAP) group (white boxes) and in the non-VAP group (boxes with gray shading). **B.** Concentrations of sTREM-1 in mBAL fluid in the VAP group on the day before VAP diagnosis (D-1), on the day of VAP diagnosis (D0) and on the day after VAP diagnosis (D+1). Central lines are medians, boxes are interquartile ranges, and brackets are total range.

No significant differences in daily sTREM-1 concentrations in mBAL fluid were observed between groups and values did not change significantly over time in either group (p = 0.5). Peak concentrations of sTREM-1 in mBAL fluid occurred in patients with VAP on POD1 (median 2414 pg/ml; range 60–11576 pg/ml) and in patients without VAP on POD5 (median 3827 pg/ml; range 693–6187 pg/ml). In the group with VAP, comparison of concentrations of sTREM-1 in mBAL fluid on the day of VAP diagnosis (D0) with those on the day before (D-1) and on the day after (D+1) the diagnosis of VAP did not show a significant difference (p = 0.24) (Figure 2). Comparison of sTREM-1 concentrations in mBAL fluid on the day of VAP diagnosis with peak sTREM-1 concentrations in mBAL fluid in the non-VAP group also did not show a significant difference (p = 0.34).

**Figure 3:**
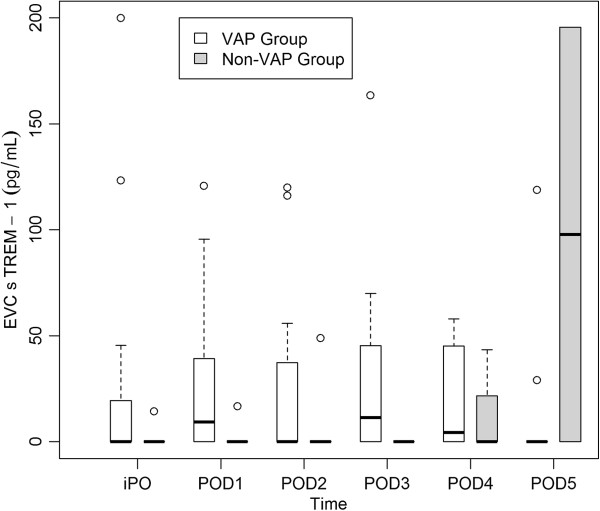
**Daily concentrations of soluble triggering receptor expressed on myeloid cells (sTREM)-1 in exhaled ventilator condensate (EVC) in the ventilator-associated pneumonia (VAP) group (white boxes) and in the non-VAP group (boxes with gray shading).** Central lines are medians, boxes are interquartile ranges, and brackets are total range.

**Figure 4:**
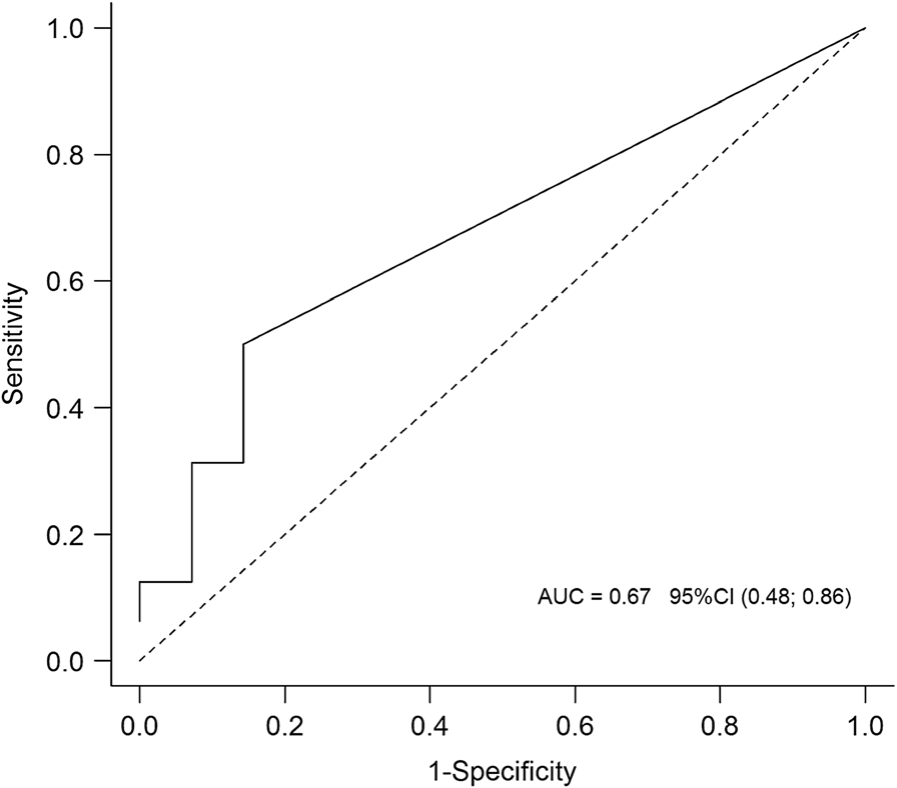
Receiver Operating Characteristic curve for soluble triggering receptor expressed on myeloid cells (sTREM)-1 levels in exhaled ventilator condensate on the day of ventilator-associated pneumonia diagnosis.

Peak sTREM-1 concentrations in EVC were observed in the VAP group on POD3 (median 11.5 pg/ml; range 0–163 pg/ml). In the non-VAP group, only two patients had detectable concentrations of sTREM-1 in EVC: one patient on POD4 (43 pg/ml) and POD5 (195 pg/ml) and the other on iPO (14 pg/ml), POD1 (17 pg/ml) and POD2 (49 pg/ml) (Figure 3). Both patients subsequently developed a surgical site infection. Twelve of 16 (75%) patients who developed VAP had sTREM-1 detected in EVC on the day of VAP diagnosis and/or the preceding day, while it was detectable in only 2 of 14 (14.3%) patients in the non-VAP group over the study period (p = 0.0013) (sensitivity 0.75, 95% CI 0.48-0.93; specificity 0.86, 95% CI 0.57-0.98; positive predictive value 0.86, 95% CI 0.57-0.98; negative predictive value 0.75, 95% CI 0.48-0.93; positive likelihood ratio (LR) 5.25, 95% CI 3.99-6.9; negative LR 0.29, 95% CI 0.22-0.39). A cut-off value of 20.58 pg/ml of sTREM-1 in EVC on the day of VAP diagnosis had a sensitivity of 0.50 (95% CI 0.26-0.75) and a specificity of 0.86 (95% CI 0.56-0.98) for the diagnosis of VAP (area under the ROC curve 0.67; 95% CI 0.48-0.86) (Figure 4). Serum and mBAL sTREM-1 yielded areas under the ROC curve 0.60 and 0.42, respectively (data not shown).

## Discussion

In the present study, we investigated the usefulness of serial measurements of sTREM-1 in serum, mBAL fluid and EVC for the diagnosis of VAP in children after congenital heart surgery. This is the first study to evaluate the diagnostic value of sTREM-1 in VAP following cardiac surgery. Our results showed that measurement of sTREM-1 in the EVC can be useful for diagnosing VAP in this population. However, sTREM-1 measurements in serum and mBAL fluid did not discriminate patients with VAP from those without VAP following cardiac surgery.

A previous study showed that plasma sTREM-1 concentrations increased after surgery in noninfected patients undergoing elective cardiac surgery with CPB and peak plasma levels of sTREM-1 overlapped with those found in patients with severe sepsis. However, no correlation was observed between sTREM-1 plasma concentrations and CPB duration or aortic cross-clamping time [15]. Our results corroborate these findings which suggest that elevated serum levels of sTREM-1 may also be related to the systemic inflammatory response to surgical stress.

In critically ill mechanically ventilated adults who developed VAP, plasma sTREM-1 levels did not change significantly over time. Nevertheless, a significant increase in mBAL fluid sTREM-1 concentration was observed. A cut-off value of 200 pg/ml combined with an increase of at least 100 pg/ml over the 6-day period before VAP was diagnosed had a sensitivity of 88% and a specificity of 84% for the diagnosis of VAP [9]. In addition, levels of sTREM-1 in BAL fluid were significantly higher in adults receiving mechanical ventilation with community-acquired pneumonia and VAP compared with patients without pneumonia [8]. In contrast, in critically ill adults with acute respiratory failure undergoing BAL for pulmonary infiltrates, the concentration of sTREM-1 in BAL fluid was not significantly greater in those with definite VAP, with the highest sTREM-1 concentrations being observed in patients with alveolar hemorrhage [21]. Accordingly, in our study, sTREM-1 concentrations in mBAL fluid were not significantly different in patients with and without VAP. Moreover, we did not observe significant changes in mBAL sTREM-1 levels from the day before to the day after VAP diagnosis. As more than 90% of patients had pulmonary congestion in the postoperative period, this could have altered sTREM-1 levels in mBAL fluid in our study.

Our data suggest that measurement of sTREM-1 in EVC may improve the accuracy of the diagnosis of VAP, which is in agreement with findings from a previous study in adults in a medical ICU, with a clinical suspicion of VAP [10]. In our study, sTREM-1 measurement in EVC samples had a good discriminative value to differentiate patients with VAP from those without VAP. Indeed, a positive test result rather than a cutoff value of sTREM-1 concentration in EVC was associated with a high probability of VAP being present (likelihood ratio of 5.2). This is especially important for patients following cardiac surgery who can have pulmonary opacities on the chest x-ray caused by multiple noninfectious conditions that may be misdiagnosed as pneumonia. In addition, EVC samples can be easily collected in a noninvasive way.

The major limitation of our study is the lack of a gold standard method for the diagnosis of VAP. However, there is currently no gold standard for the diagnosis of VAP in the clinical setting [22]. Other limitation is the lack of measurements of sTREM-1 concentrations before surgery to determine the baseline patients’ levels.

## Conclusion

The detection of sTREM-1 in EVC samples may be useful for the diagnosis of VAP after heart surgery.

## Abbreviations

BAL: Bronchoalveolar lavage; CDC: Centers for Disease Control and Prevention; EVC: Exhaled ventilator condensate; iPO: Immediate postoperative; mBAL: Modified non-bronchoscopic bronchoalveolar lavage; PICU: Pediatric intensive care unit; POD: Postoperative day; TREM-1: Triggering receptor expressed on myeloid cells-1; sTREM-1: Soluble triggering receptor expressed on myeloid cells-1; VAP: Ventilator-associated pneumonia.

## Competing interests

The authors have no conflicts of interest to disclose.

## Authors’ contributions

AM contributed with study design, data collection and manuscript writing. AC contributed with study design, statistical analysis, manuscript writing, and was the study coordinator. Both authors read and approved the final manuscript.

## Pre-publication history

The pre-publication history for this paper can be accessed here:

http://www.biomedcentral.com/1471-2261/13/107/prepub
